# RIPK1 signaling pathways: implications for autoimmune and neuroinflammatory diseases

**DOI:** 10.3389/fimmu.2025.1642593

**Published:** 2025-09-12

**Authors:** Abigail Pajulas, Jonathan T. Sims, Eric P. Hanson, Andrew C. Vendel

**Affiliations:** ^1^ Immunology Research, Eli Lilly and Company, San Diego, CA, United States; ^2^ Immunology Research, Eli Lilly and Company, Indianapolis, IN, United States

**Keywords:** autoimmunity, neuroinflammation, cell death, immunology, RIPK1, inflammatory diseases

## Abstract

Receptor-interacting protein kinase 1 (RIPK1), which regulates cell death and survival pathways, is a promising therapeutic target for the treatment of several autoimmune, inflammatory, and neurodegenerative diseases, having important roles in inflammation, apoptosis, and necroptosis. In this review, we describe recent insights that help elucidate the molecular mechanisms of signaling pathways reliant on RIPK1, focusing on its scaffolding function and kinase activity. We emphasize the cell type-specific effects of RIPK1, characterizing its role in necroptosis, immune cell regulation, and tissue-specific responses. Lastly, we present the relevance of RIPK1 in autoimmune and inflammatory diseases, while highlighting the clinical landscape for RIPK1-targeting therapies. All together, this review aims to present recent findings pertaining to RIPK1 signaling and discuss its potential as a therapeutic target in diseases.

## 1 Introduction

**Figure 1:**
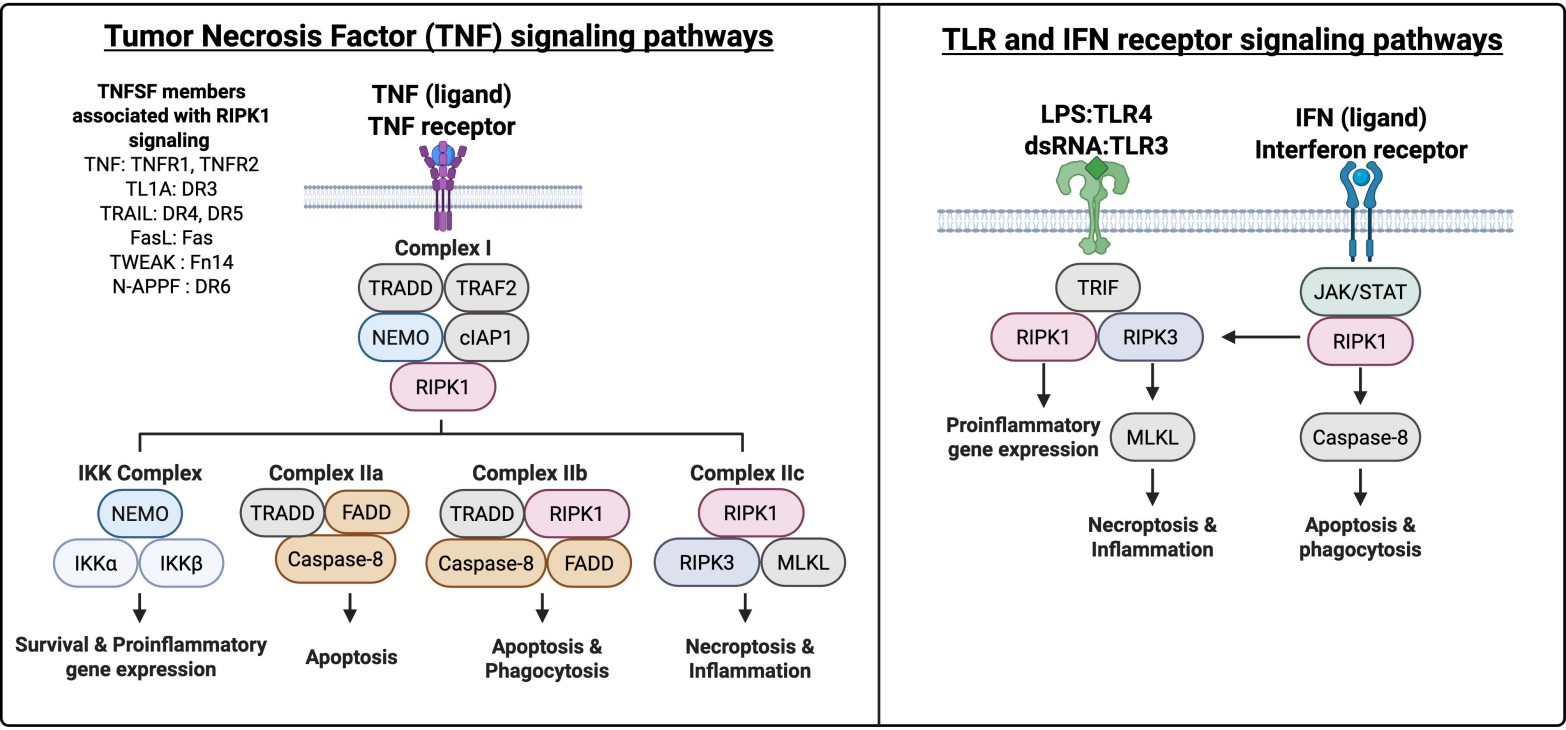
Signaling pathways involving RIPK1. RIPK1 participates in the regulation of several signaling pathways that control survival, apoptosis, necroptosis, and inflammation. RIPK1 can function as a kinase and a scaffold protein to contribute to canonical NF-κB signaling in response to TNF/TNF superfamily (TNFSF) stimulation. Additionally, RIPK1 utilizes its kinase activity to influence immune responses to interferons (IFN), toll-like receptor (TLR) ligands, and TNF/TNFSF ligands, including Akt and AP-1/JNK signaling to regulate cell growth and stress responses.

Receptor-interacting serine/threonine-protein kinase 1 (RIPK1) is involved in the regulation of signaling pathways from various cell receptors that control survival, apoptosis, and inflammation (1, 2). RIPK1 consists of 671 amino acids and has a molecular weight of approximately 76 kDa (2). RIPK1 modulates inflammation and cell death in response to cytokines, RNA, and pathogen toxins such as lipopolysaccharide (LPS) through its kinase activity and scaffolding function (**Figure 1**) (3).

RIPK1 has three essential domains including a serine/threonine kinase domain and a death domain, separated by an intermediate domain (1). The N-terminal kinase domain is involved in downstream cell death signaling pathways that involve RIPK3, mixed lineage kinase domain-like protein (MLKL), and caspase-8 (4, 5). The kinase domain contains a unique allosteric pocket that is suited for small-molecule inhibitors allowing for specific inhibition of RIPK1’s kinase activity while retaining its scaffolding function (4). The C-terminal death domain of RIPK1 can interact with the death domain of receptors such as Fas, tumor necrosis factor (TNF) receptor 1 (TNFR1), death receptors, Toll-like receptors (TLR), interferon (IFN) receptors (IFNR), and TNF-related apoptosis-inducing ligand (TRAIL) to induce cell death (4). Lastly, the intermediate domain contains the receptor-interacting protein homotypic interaction motif (RHIM), which interacts with RIPK3 forming a protein complex that can modulate nuclear factor kappa-light-chain-enhancer of activated B cells (NF-κB) activation and other signaling interactions (4). Furthermore, when activated, RIPK1 can translocate to the nucleus where it interacts with the Brahma-related gene 1 (BRG1)/Brahma gene (BRM)-associated factor (BAF) complex, the mammalian homolog of the switch/sucrose nonfermentable (SWI/SNF) complex in yeast, to promote chromatin remodeling and transcription (6).

In this review, we first highlight signaling pathways associated with RIPK1. We then discuss cell type-specific roles of RIPK1, highlighting key signaling pathways associated with various cell responses. Finally, we discuss the function of RIPK1 in disease and examine the different therapeutic approaches related to RIPK1.

## 2 Receptors and signaling pathways involving RIPK1

RIPK1 acts as a critical molecular switch, balancing cell survival and death in response to environmental cues. The distinct functions of RIPK1 can be attributed to its roles as both a kinase and a scaffold. For example, RIPK1 contributes as a scaffold protein to engage the canonical NF-κB signaling pathway in response to TNF stimulation, although RIPK1 is not always required (4, 7, 8). Alternatively, RIPK1 utilizes its kinase activity to influence immune responses including Akt activation to regulate cell growth and activator protein-1 (AP-1)/Jun N-terminal kinase (JNK) signaling to regulate the stress response (9, 10). Depending on the context and signals received, RIPK1 can contribute to controlled cell death decisions through apoptosis or through necroptosis, a proinflammatory form of death (2) (**Figure 1**). By integrating signals from various stimuli such as cyclin-dependent kinases, cytokine receptors, and inducers of autophagy, RIPK1 maintains cellular homeostasis and regulates immune responses effectively, ensuring appropriate cellular outcomes (11–14). However, because characterizing the relative activities of these various signaling pathways *in vivo* is challenging, there remain significant knowledge gaps regarding the cell type-specific physiologic functions of RIPK1. While TNF signaling pathways have been extensively studied using cell culture and murine models (15), the contributions of modulating pathways in different cell types and disease contexts may be crucial for developing targeted therapies that leverage RIPK1’s signaling networks. This is particularly relevant in instances in which pathways that involve RIPK1 can still be activated in the absence of RIPK1.

### 2.1 TNF receptor superfamily members: TNFR1, Fas, DR3

When TNF binds TNFR1, TNFR1-associated death domain protein (TRADD) is recruited to the cytoplasmic tail of TNFR1. TRADD forms a prosurvival complex known as TNFR1 signaling complex I with RIPK1, TNFR-associated factor 2 (TRAF2), and cellular inhibitor of apoptosis proteins 1 and 2 (cIAP1/2) (4) (**Figure 1**). TRAF2/cIAP1/2 promotes ubiquitination of RIPK1 by recruiting a heterotrimeric ligase, linear ubiquitin chain assembly complex (LUBAC), which consists of the three proteins heme-oxidized IRP2 ubiquitin ligase 1 (HOIL1), HOIL1-interacting protein (HOIP), and SHANK-associated RH domain-interacting protein (SHARPIN) to allow for protein ubiquitination (16, 17). This process stabilizes RIPK1 through ubiquitination and triggers both prosurvival and proinflammatory responses via mitogen-activated protein kinase (MAPK) and NF-κB pathways (18, 19). TNF-induced NF-κB nuclear translocation leads to gene activation and expression of proinflammatory cytokines and prosurvival proteins such as cIAP2 and caspase-8 inhibitor cellular FLICE inhibitor protein (cFLIP), ultimately promoting cell survival and inflammation (20) (**Figure 1**).

Post-translational modification of RIPK1 and other components within the TNFR1 signaling complex can initiate a transition from prosurvival/inflammatory responses to cell death mechanisms such as apoptosis or necroptosis (19, 21–23). Distinct ubiquitin modifications on E3 ligases cIAP and LUBAC result in their degradation or exclusion from the TNFR1 signaling complex. This allows TRADD to recruit RIPK1 and Fas-associated death domain (FADD) forming complex IIb, referred to as the ripoptosome complex, leading to caspase-8-mediated apoptosis (24) (**Figure 1**). Whereas the kinase activity of RIPK1 can drive programmed cell death, the scaffolding function can be protective, as RIPK1 deficiency leads to FADD and caspase-8 mediated apoptosis, both during development and in response to Fas (TNFR superfamily 6 [TNFRSF6]) signaling as observed in auto-reactive B lymphocytes within germinal centers (25) (**Figure 1**). In addition to modifications in RIPK1, caspase-8 can also be regulated by post-translational modifications which can influence its activity, stability, and protein interactions. Dysregulation of TNF/TNF superfamily (TNFSF) signaling and reduced caspase-8 activity, as observed in certain cancers, can result in elevated levels of RIPK1 that can shift and change the cell’s fate from apoptosis to necroptosis, enhancing inflammation (26). The activation of RIPK1-dependent necroptosis is caspase-independent and involves RIPK1/RIPK3-dependent activation of MLKL, resulting in membrane permeabilization and subsequent release of proinflammatory cytokines and chemokines upon necroptosis-mediated cell death (27, 28). Although inhibiting TNF has been effective (22, 29), targeting certain TNFRSF members like Fas has proven problematic, resulting in hepatocyte apoptosis and liver toxicity. This indicates the need to proceed cautiously in the identification and development of other more selective and tissue-specific targets within this family (30).

Death receptor 3 (DR3, TNFRSF25) is also part of the TNFRSF and is expressed on lymphocytes: innate lymphoid cells, T cells, and B cells. It serves as a receptor for the TNF-like protein 1A (TL1A, TNFSF15) whose expression is induced by proinflammatory conditions (31). The interactions between TL1A/DR3 initiate downstream signaling cascades analogous to those of TNF/TNFR1 through its death domain, forming complex I (**Figure 1**). Despite the similarities in downstream signaling among TNFSF members, expression of TL1A/DR3 is tightly regulated and can selectively regulate different lymphocyte subsets such as effector T cells and T regulatory cells under inflammatory conditions to enhance effector function (31). TL1A inhibitors have shown promise in mouse models for multiple sclerosis (MS), inflammatory bowel disease (IBD), and rheumatoid arthritis (RA), leading to further research characterizing TL1A’s role in inflammatory diseases to better anticipate potential side effects (32). Although therapies targeting TL1A are currently undergoing phase 3 clinical trials, further research is required to ascertain the potential of RIPK1 inhibitors as a viable treatment option for TL1A-related conditions.

### 2.2 Type I and type II interferons

In response to microbial or endogenous DNA or RNA, pattern recognition receptors such as cGAS-STING, RIG-I/MDA5-MAVS, and TLR3/4 initiate signaling pathways that can upregulate type I IFNs, particularly IFNα and IFNβ (33) (**Figure 1**). These cytokines are crucial for both innate and adaptive immunity, providing antiviral defense, modulating immune responses, aiding in clonal expansion and activation of memory CD8 T cells, and enhancing humoral immunity by promoting isotype switching and pDC maturation (33) (**Figure 1**). RIPK1 plays a role in regulating the production and signaling of type I and type II IFNs. In the absence or inhibition of caspase-8, RIPK1 associates with TANK-binding kinase 1 (TBK1), which leads to type I IFN production and increased antiviral resistance, indicating a role for RIPK1 in antiviral defense mechanisms (34). These proinflammatory mediators triggered by RIPK1 signaling often act independently of RIPK3, highlighting RIPK1’s distinct role in initiating downstream pathways (35).

As alluded to earlier, RIPK1 is also associated with downstream IFN signaling pathways. During *Salmonella* infections, type I IFNs can trigger RIPK1 and RIPK3 aggregation to induce MLKL-dependent necroptosis (36, 37). Type II IFNs have a similar signaling capacity in which IFNγ can induce RIPK1-dependent or RIPK1-independent MLKL-mediated necroptosis (38). During RIPK1-independent necroptosis, Z-form nucleic acid binding protein 1 (ZBP1) can directly bind and activate RIPK3 to phosphorylate MLKL (39). However, RIPK1 can influence this response by competing with ZBP1 for binding to RIPK3, thereby preventing the formation of the ZBP1/RIPK3 complex (40). This suggests that RIPK1 plays a protective role against IFN-induced toxicity in cells. Although RIPK1 kinase-mediated necroptosis can function as a defense mechanism during infections, necroptosis contributes to inflammation seen in murine models of inflammatory diseases (4, 23, 41). Necroptosis can occur through RIPK1-dependent and RIPK1-independent pathways, implying that combination therapy might be necessary to effectively target both pathways.

### 2.3 Viral RNA/TLR3 and LPS/TLR4

TLR3 and TLR4 recognize pathogen-associated molecular patterns (PAMPs) and damage-associated molecular patterns (DAMPs). TLR3 binds to double-stranded RNA, typically associated with viral infections, leading to the production of type I IFNs and proinflammatory cytokines. This activation can exacerbate autoimmune diseases such as systemic lupus erythematosus and MS. TLR4 detects LPS from gram-negative bacteria and DAMPs from stressed cells, triggering inflammatory pathways that lead to chronic inflammation and tissue damage. TLR4 activation is associated with autoimmune diseases such as RA and systemic sclerosis.

Upon recognizing PAMPs or DAMPs, TLRs initiate downstream RIPK1-dependent or RIPK1-independent signaling pathways (11, 42) (**Figure 1**). In the RIPK1-dependent pathway, TRIF serves as a key adaptor protein that recruits RIPK1 to the TLR signaling complex, promoting activation of MAPK and canonical NF-κB signaling, leading to transcription of proinflammatory cytokines such as TNF and interleukin (IL)-1β (42, 43) (**Figure 1**). Moreover, Toll/IL-1 receptor domain-containing adapter protein (TRIF)-TLR4 interactions can trigger the formation of necrosomes composed of RIPK1 and RIPK3 that can leverage NOD-, LRR- and pyrin domain-containing protein-3 (NLRP3) inflammasome-mediated proinflammatory cytokine production such as pro-IL-1β, pro-IL-18, and gasdermin D (41, 44, 45). This pathway is further modulated by ZBP1, which enhances RIPK1 ubiquitination and delivery to TRIF (46). In contrast, RIPK1-independent signaling is mediated through direct recruitment of RIPK3 by TRIF, as shown in TLR-induced necroptosis in macrophages, where RIPK3 activation occurred independent of TNF or RIPK1 (47). ZBP1 can trigger necroptosis by interacting with RIPK3 through RHIM-RHIM binding without involving RIPK1 (48). These parallel pathways highlight the context-dependent roles of RIPK1 in immune signaling, in which it can act as both a scaffold for inflammatory signaling and a regulator of cell death, depending on the upstream stimuli and cell context. This process involves the activation of the NLRP3 inflammasome, which consists of NLRP3, caspase-1, and adaptor apoptosis-associated speck-like protein (ASC) and is capable of cleaving inflammatory cytokines.

### 2.4 Pathogens

Pathogens have developed specialized mechanisms to manipulate key regulators of inflammation and cell death such as RIPK1 to evade host immune defenses. One major mechanism involves disrupting cytokine production initiated by TNF/TNFR1 and TLR signaling pathways, which are crucial for an effective immune response. Pathogens can do this by disrupting cIAP-LUBAC–dependent NF-κB signaling, which not only limits cytokine production but also decreases cFLIP levels, ultimately shifting the balance toward cell death instead of survival (49).

Pathogens may exploit necroptosis by creating conditions that inhibit caspase-8 activity, thereby preventing apoptosis. They can reduce the ubiquitination of RIPK1, which stabilizes RIPK1-RIPK3 complex formation, promoting necroptotic cell death (49). Some pathogens might also directly impair the function of cIAPs or LUBAC, further enhancing RIPK1-mediated necroptosis (49). This approach enables pathogens to evade immune responses and influence host cell death pathways by releasing factors that regulate cell death and survival mechanisms for their benefit.

Bacteria have evolved to inhibit RIPK1 signaling; for example, *Porphyromonas gingivalis* has been shown to specifically cleave RIPK1 via its lysine-specific protease Kgp in human endothelial cells (50). *P. gingivalis* can directly induce the proteolysis of RIPK1 without needing to activate innate immune signaling pathways or caspase-dependent apoptotic pathways (13). Similarly, *Salmonella* Typhimurium exploits inflammasome-mediated RIPK1-dependent necroptosis to facilitate systemic dissemination of (51). In contrast, some pathogens trigger RIPK1 activation as part of the host’s defense strategy. *Yersinia pestis* utilizes effector proteins to block signaling pathways associated with cytokine production (52). In response, host cells activate RIPK1, which complexes with caspase-8 and RIPK3, leading to gasdermin D- and E-mediated pyroptosis in macrophages and neutrophils (52). This process of cell death facilitates IL-1β release and contributes to limiting bacterial dissemination. Thus, while some bacteria suppress RIPK1 to evade immune detection, other inadvertently trigger RIPK1-driven cell death, which the host leverages as a protective mechanism.

Viral pathogens also manipulate RIPK1 signaling. In RNA virus infections, RIPK1 forms a complex with RIPK3 that activates the GTPase DRP1, leading to mitochondrial damage, reactive oxygen species (ROS) production, and activation of the NLRP3 inflammasome (53). This pathway leads to the secretion of proinflammatory cytokines such as IL-1β and IL-18 (53). Notably, inactivation of RIPK1 or RIPK3 significantly impairs caspase-1 cleavage and cytokine release during RNA virus infections, but not without DNA virus–induced NLRP3 inflammasome activation, suggesting pathogen-specific modulation of RIPK1 pathways (53). Moreover, recent findings show that in the absence of caspase-8, RIPK1 can aberrantly interact with TBK1 to drive type I IFN production, enhancing resistance to norovirus but also contributing to lymphadenopathy and systemic inflammation (34). This highlights RIPK1’s dual role in antiviral defense and immunopathology, depending on the context of infection and regulatory balance.

Collectively, these findings underscore the association between pathogens and RIPK1 signaling, illustrating how pathogens influence host cell fate, evade immune detection, and enhance their own survival. Understanding these interactions offers potential therapeutic avenues for controlling infection-induced inflammation and tissue damage.

## 3 Biological functions of RIPK1

Depending on the cell type and the surrounding immune environment, RIPK1 can either induce cell death or trigger inflammation. Here, we explore cell type-specific RIPK1 functions in the hope of better understanding its role in homeostasis.

### 3.1 Lymphocytes

RIPK1 has been studied for its role in regulating T and B lymphocyte activation, survival, and cell death in response to TNF and members of the TNFSF. Given that lymphocytes can contribute to initiating, promoting, and sustaining inflammation, RIPK1 is uniquely positioned to regulate the inflammatory responses. In murine models, conditional RIPK1 deficiency in lymphocytes can enhance mTORC1-dependent prosurvival gene transcription and inflammatory cytokine production, suggesting that RIPK1 in T cells plays a critical role in controlling inflammatory disease (54). This is consistent with human data showing higher naïve T lymphocyte frequencies in RIPK1-deficient patients, but it’s unclear if RIPK1 deficiency is a kinase- or scaffold-specific mechanism (55, 56). In contrast, inhibitors of RIPK1 kinase activity in B cells have the potential to prevent B cell death (57). This highlights the importance of considering the effects of targeting RIPK1 on lymphocyte activity in a disease-specific context. Notably, RIPK1-deficiency in naïve CD4+ T cells displayed heightened sensitivity to TNFR1-induced apoptosis upon TNF stimulation (13). This observation suggests that signaling pathways capable of activating lymphocytes may concurrently render these cells more vulnerable to cell death through an alternate signaling pathway within the same cell. This may be due in part to decreased NF-κB phosphorylation in response to TNF as observed in RIPK1-deficient Jurkat cells (55). It is essential to delineate the specific roles of RIPK1 within the diverse signaling pathways illustrated in **Figure 1**.

### 3.2 Innate immune cells

RIPK1 is a key mediator in monocyte, macrophage, and dendritic cell signaling pathways, including TNFR, TLR, and IFNR. RIPK1-mediated signaling pathways facilitate the clearance of infections by promoting inflammatory responses and cell death mechanisms that limit pathogen spread. For instance, beyond cytokine secretion in response to TLR and IFNR signaling, RIPK1 can induce necroptosis in myeloid cells which intensifies inflammation by releasing DAMPs like HMGB1, IL-1α, and IL-1β (12, 37, 41, 58, 59). Importantly, RIPK1 maintains cellular balance by regulating downstream signaling interactions. RIPK1 deficiency may cause IAP degradation, making dendritic cells more susceptible to necroptosis (60). This occurs as RIPK3 complexes with factors like MLKL to form a necrosome or complex IIc, inducing cellular necroptosis and releasing proinflammatory mediators (60). Notably, these findings in both mouse and human cells support a role for RIPK1 to regulate RIPK3 activity while also supporting RIPK3-independent utility. Indeed, RIPK1 kinase activity can enhance murine dendritic cell activation even in the absence of RIPK3 and caspase-8 (12). Collectively, these findings indicate that RIPK3 and caspase-8 are dispensable for RIPK1 kinase-mediated inflammatory responses in certain inflammatory conditions.

Like myeloid cells, neutrophils utilize RIPK1 to regulate cell death and effector functions. *In vitro* mouse cell cultures showed that inhibition of RIPK1 greatly reduced LPS-induced ROS and TNF generation and blocked the NF-κB pathway without causing neutrophil cell death, suggesting that RIPK1 has dual roles in modulating cellular metabolism to control cellular responses (61). This is not surprising given that ROS and RIPK1 are closely associated with the NF-κB signaling pathway, which is intricately connected to inflammatory responses and oxidative stress (23). Indeed, LPS/TLR4 signaling in neutrophils allows for increased RIPK1 phosphorylation and RIPK1/RIPK3/MLKL necrosome complex formation (62). As alluded to earlier, neutrophil death and neutrophil extracellular trap (NET) formation may involve the signaling pathway defining necroptosis downstream of ROS production. Notably, activation of RIPK1 in human neutrophils was important for MLKL phosphorylation that can promote NET formation (63).

### 3.3 Epithelial cells

Epithelial cells use RIPK1 signaling to manage necroptosis and maintain barrier functions. RIPK1 employs a kinase-independent scaffolding mechanism to manage epithelial cell apoptosis and necroptosis *in vivo*, which is crucial for maintaining physiological homeostasis in the intestine and skin (3). In epithelial cells lining the gut, RIPK1 has dual opposing roles. For instance, intestinal epithelial cell (IEC)-specific RIPK1 knockout caused IEC apoptosis, villus atrophy, loss of goblet and Paneth cells and premature death in mice, suggesting that RIPK1 in epithelial cells promotes homeostasis (3). Epithelial cells are capable of undergoing necroptosis via an alternative pathway that does not require RIPK1. In this process, RHIM-containing adaptors such as TRIF or ZBP1 activate RIPK3 directly, resulting in MLKL-mediated membrane disruption and the induction of inflammatory responses, as demonstrated in models of MS (64). Another study observed that RIPK1 deficiency sensitized IECs to TNF-induced apoptosis independently of any defect in NF-κB activation, whereas sensitization of RIPK1-deficient mouse embryonic fibroblasts was associated with defective NF-κB activation (65). This demonstrated that RIPK1 can contribute to a proinflammatory role downstream of TNF signaling in IECs. Surprisingly, TNF-induced death of IKKβ(EE)-expressing human and mouse IECs induces RIPK1 kinase activity, as opposed to scaffolding activity, to control epithelial cell death (66). This seemingly paradoxical activity further reinforces the idea that RIPK1 may display divergent phenotypes depending on whether it functions as a kinase or a scaffold, with outcomes influenced by cell type and tissue environment. RIPK1 can also contribute to murine fibroblast cell death. Notably, both type I and type II IFNs can activate RIPK1-RIPK3-dependent necroptosis in murine fibroblasts even in the presence or absence of adaptor proteins: FADD and caspases (67). Further studies are needed to better understand the exact role of RIPK1 in mucosal epithelial cells to define situations where inhibiting or enhancing RIPK1 would be beneficial from a therapeutic standpoint.

### 3.4 Nervous system-associated cells

In the mature nervous system, RIPK1-dependent necroptosis plays a central role in determining cell fate – either survival or death – in response to inflammatory signals such as TNF, Fas, and TRAIL (68). In addition to its role as a primary regulator of necroptotic cell death, RIPK1 also regulates neuroinflammation. In microglia under physiological conditions, RIPK1 activation leads to transcription of neuroinflammatory genes amplifying the inflammatory response within the central nervous system (CNS) to support cellular clearance and immune surveillance (64, 69). One key mechanism involves the modulation of *CST7*, which encodes cystatin F, an endogenous inhibitor of lysosomal cathepsins (70). RIPK1 helps maintain cystatin F levels to support lysosomal proteolytic activity and microglial clearance functions. However, aberrant RIPK1 activation leads to overexpression of cystatin F, resulting in impaired cathepsin activity and lysosomal dysfunction (70). This disruption compromises microglial homeostasis and contributes to the accumulation of neurotoxic debris that can amplify the inflammatory response. Impairment of the lysosomal degradation pathway further disrupts microglial equilibrium and exacerbates the inflammatory milieu, which may perpetuate a cycle of chronic neuroinflammation and progressive neurodegeneration. Accordingly, RIPK1 serves dual roles in CNS pathology – both initiating cell death and sustaining inflammation.

## 4 RIPK1 role in diseases

RIPK1 is being studied as a potential therapeutic target for conditions such as ischemia-reperfusion injuries, atherosclerosis, RA, IBD, retinal damage, and MS. While inflammation is typically associated with necroptosis, research suggests that RIPK1 may regulate inflammation independent of cell death (47, 48, 64). It is essential to assess whether inflammatory diseases, where RIPK1’s role in cell death is suspected to be sparse, might involve regulating inflammation independently of cell death.

### 4.1 Inborn errors of immunity

RIPK1 variants (D324V and D324H) prevent cleavage by caspase-8 leading to increasing susceptibility to RIPK1 activation, apoptosis, and necroptosis. Consequently, patients with these variants exhibit heightened inflammatory signaling and produce more inflammatory molecules compared to unaffected individuals, which indicates RIPK1 causality in humans (71). Patient P1 showed a positive clinical response to IL-6 blockade using tocilizumab, a monoclonal antibody targeting IL-6R, consistent with outcomes observed in another patient with RIPK1 variant-associated autoimmunity (71, 72). These results suggest that the symptoms may be associated with increased proinflammatory cytokine expression such as IL-6. These results support a non-redundant role for RIPK1 kinase activity that can impact both apoptosis and necroptosis, but also transcriptional production of proinflammatory cytokines such as IL-6 (71). Complete loss of RIPK1 in humans resulted in immunodeficiency and diarrhea; however, missense mutations within the death domain of RIPK1 impacting kinase activity resulted in IBD-like conditions (55). Moreover, loss of function of RIPK1 is also associated with RA-like symptoms. Patients with compound heterozygous RIPK1 variants (K377E/R390G) exhibit recurrent fevers, lymphadenopathy, and skin rashes (73).

In addition to RIPK1 variants, variants of proteins that can regulate RIPK1 ubiquitination and phosphorylation, such as variants in the *TNFAIP3* gene, which encodes the protein A20, are linked with autoimmunity and inflammation, and likely also contribute to irregularities of RIPK1 function (74, 75). A20 functions as a negative feedback regulator of NF-κB and apoptosis (74). It is known to associate with complex IIb to bind linear ubiquitin for stabilizing scaffolding within the ripoptosome, supporting the critical role for A20 in supporting RIPK1 kinase activity (74). However, the role of A20 in ripoptosome regulation is complex- and context-dependent. While A20 can promote RIPK1 activation in some settings, recent findings demonstrate that in T cells, A20 plays a protective role by inhibiting RIPK1-mediated apoptosis (76). This again underscores the importance of tissue-specific differences in RIPK1 signaling and the need to consider cellular context when targeting these pathways therapeutically.

### 4.2 Rheumatoid arthritis

**Figure 2:**
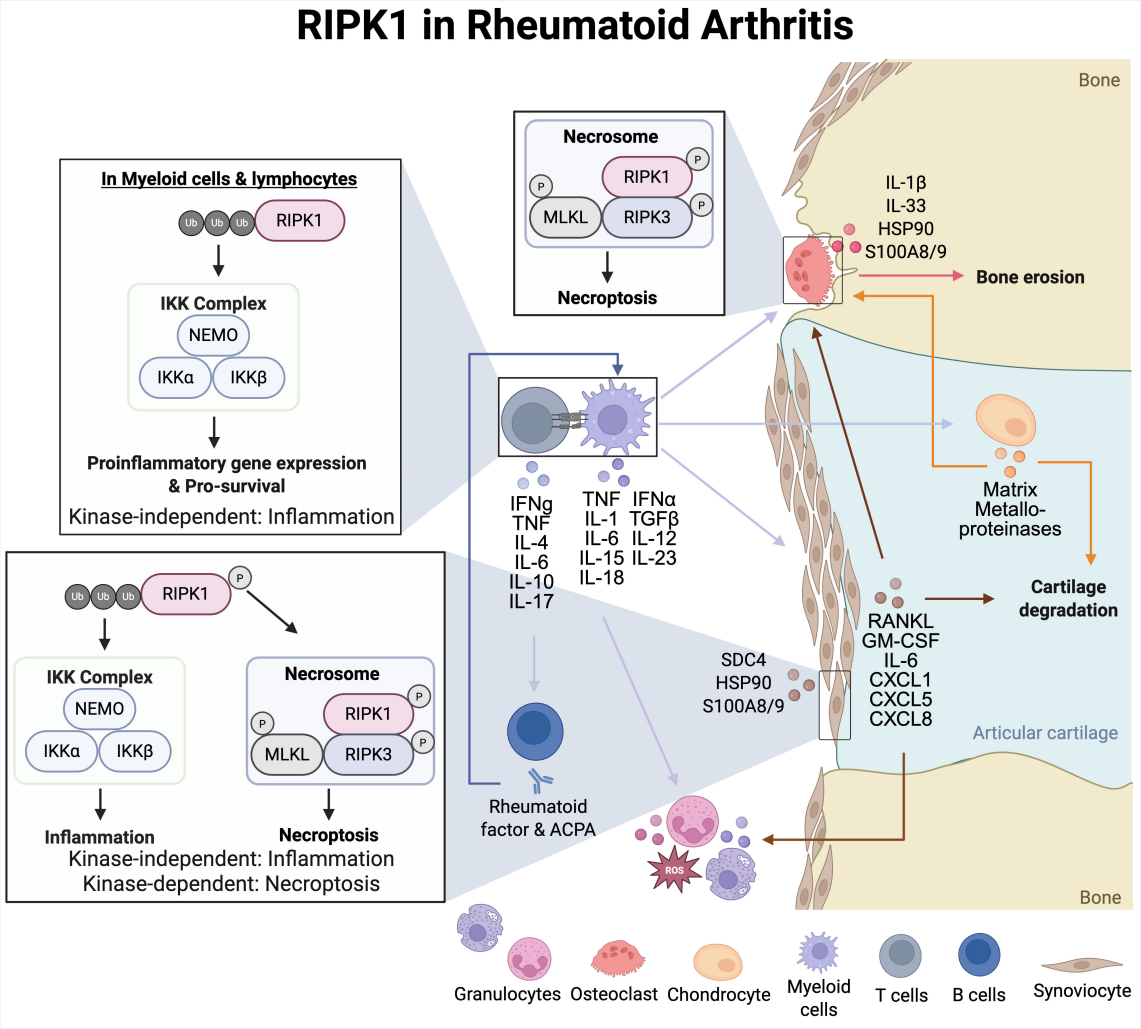
RIPK1 in rheumatoid arthritis. Myeloid cells including macrophages and dendritic cells present antigens to T cells, causing T cell activation and proliferation. Activated T cells and myeloid cells produce inflammatory cytokines to contribute to join inflammation. A crucial effector cytokine in the initiation of RA pathogenesis is tumor necrosis factor, which affects synoviocytes, macrophages/monocytes, and osteoclasts. Joint degradation results from macrophage recruitment and inflammatory cytokine secretion, while bone erosion is due to osteoclast activity and reduced collagen secretion by synoviocytes. Joint damage results in granulocyte recruitment of neutrophils and mast cells into the joint space, causing hyperplasia and angiogenesis of the synovial membrane that can lead to diminished joint mobility.

RA is a progressive autoimmune disease that causes severe bone loss and joint inflammation. In RA, innate immune cells such as monocytes, dendritic cells, and mast cells interact with the adaptive immune cells (T cells, B cells, and plasma cells) within the synovial membrane to contribute to inflammation in the joints (**Figure 2**). Mechanistically, RIPK1 biallelic variants can impair RIPK1 ubiquitination leading to the loss of RIPK1 protein expression, which can ultimately suppress the formation of the TNFR1 signaling complex and reduced NF-κB activation, resulting in arthritis (73). Alternatively, RIPK1 inhibition in a murine collagen-induced arthritis model was able to reduce proinflammatory cytokine expression of IL-17, IL-1β, IL-6, and TNFα, suggesting that RIPK1 inhibition has the potential to impact myeloid and T cell responses (77). It is important to note that the responses observed in murine models seldom mirror clinical outcomes, especially since the induction of arthritis in the murine model does not accurately recapitulate genetic abnormalities associated with RIPK1 in humans. Currently, there is an ongoing clinical trial in RA populations to better characterize the therapeutic potential of targeting RIPK1 (**Table 1**).

**Table 1:** RIPK1 inhibitors in clinical trials.

Name	Company	Indication	Phase	Clinical trial #	Status	Reference
ABBV-668	AbbVie	Ulcerative colitis (moderate to severe)	Phase 2	NCT05570006	Completed 2024-12-23	
AC-003	Accropeutics	N/A	Phase 1	CTR20222268 (China)	Completed 2023-05-19	
		Acute graft-versus-host disease	Phase 1	CTR20233988 (China)	Recruiting	
DNL104	Denali Therapeutics	N/A	Phase 1	NTR6257 (Netherlands)	Terminated following the detection of off-target liver toxicity signals	(97)
DNL747 (SAR443060)	Denali Therapeutics/Sanofi	Amyotrophic lateral sclerosis	Phase 1	NCT03757351	Terminated due to change in Sanofi’s development strategy	(93)
		Alzheimer’s disease	Phase 1	NCT03757325	Completed 2019-12-05	(93)
DNL758 (SAR443122)	Denali Therapeutics/Sanofi	COVID-19	Phase 1	NCT04469621	Completed 2020-10-23	(98)
		Cutaneous lupus erythematosis	Phase 2	NCT04781816	Completed 2023-06-26	
		Ulcerative colitis (moderate to severe)	Phase 2	NCT05588843	Recruiting	
DNL788 (SAR443820)	Denali Therapeutics/Sanofi	Brain penetrant	Phase 1	NCT05795907	Completed 2021-07-20	(99)
		Amyotrophic lateral sclerosis	Phase 2	NCT05237284	Terminated as Part A did not meet the primary endpoint.	
		Multiple sclerosis	Phase 2	NCT05630547	Terminated as did not meet primary and key secondary endpoints	
GDC-8264	Genentech	N/A	Phase 1	EudraCT: 2019–002613-19	Completed 2021-10-13	(100)
		Acute graft-versus-host disease	Phase 1	NCT05673876	Terminated for business reasons	
		Cardiac surgery-associated acute kidney injury and major adverse kidney events	Phase 2	NCT06602453	Recruiting	
GFH312	GenFleet Therapeutics	N/A	Phase 1	NCT04676711	Completed 2022-10-31	(101)
		Peripheral artery disease	Phase 2	NCT05618691	Withdrawn for reasons of adjusted clinical development strategy	
GSK2982772	GlaxoSmithKline	Plaque psoriasis (moderate to severe)	Phase 1	NCT04316585	Completed 2021-10-12	(80)
		Plaque psoriasis (mild to moderate)	Phase 2	NCT02776033	Completed 2018-01-04	(81)
		Rheumatoid arthritis (moderate to severe)	Phase 2	NCT02858492	Completed 2018-10-22	(92)
		Ulcerative colitis (active)	Phase 2	NCT02903966	Completed 2019-06-17	(82)
GSK3145095	GlaxoSmithKline	Pancreatic ductal adenocarcinoma and other selected tumors	Phase 1	NCT03681951	Terminated following an internal review of the company’s research and development portfolio	(102)
R552 (LY3871801)	Rigel Pharmaceuticals/Eli Lilly	N/A	Phase 1	EudraCT: 2019-002520-32	Completed 2022-03-11	(84)
		Rheumatoid arthritis (active moderate to severe)	Phase 2	NCT05848258	Recruiting	
SIR1-365	Sironax	COVID-19	Phase 1	NCT04622332	Completed 2021-11-27	(103)
		Chronic prostatitis/chronic pelvic pain syndrome	Phase 1	ACTRN12621000745842p (Australia)	Submitted 2021-04-13 Not yet approved	(104)
SIR2446M	Sironax	N/A	Phase 1	ACTRN12621001621808 (Australia)	Completed 2023-04-28	(105)
SIR9900	Sironax	N/A	Phase 1	ACTRN12623000696695 (Australia)	Completed 2024-01-23	(106)
SIR9900	Sironax	N/A	Phase 1	ACTRN12623000790640 (Australia)	Completed 2023-12-28	(106)

N/A, not applicable, tested in healthy adults.

### 4.3 Psoriasis

**Figure 3:**
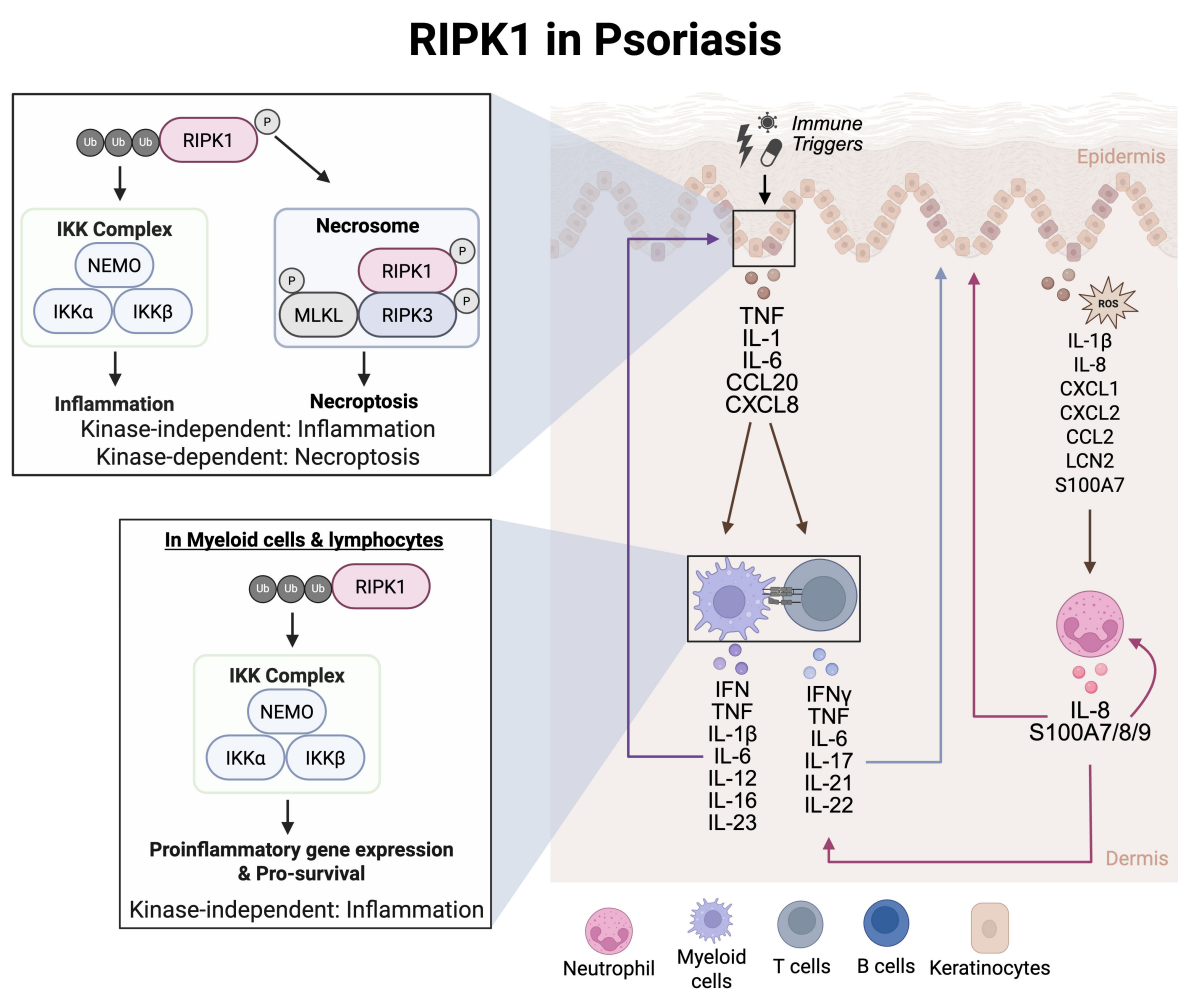
RIPK1 in psoriasis. Myeloid cells such as dendritic cells can respond to IFN stimulation to secrete IL-12 and IL-23. IL-12 induced Th1 cell differentiation while IL-23 induced Th17 cell differentiation in naïve T cells. The T cells then contribute to the cytokine milieu by secreting IFNγ, TNF, IL-22, and IL-17, These secreted cytokine and chemokines activate intracellular signaling pathways in keratinocytes to regulate gene transcription of cytokines and chemokines. This leads to the recruitment of neutrophils, initiating an inflammatory signaling cascade that involves excessive feed-forward activation of the immune system.

RIPK1 dysregulation contributes to psoriasis pathogenesis, but the therapeutic impact remains under investigation. One of the key effector cell types in psoriasis are neutrophils (**Figure 3**). Neutrophils, at a cellular level, are impacted by RIPK1. Low levels of RIPK1 protein expression can be detected in monocytes and neutrophils from peripheral blood of psoriasis patients (78). In isolated psoriasis neutrophils, RIPK1 and caspase-8 mRNA were downregulated while RIPK3 and MLKL mRNA were elevated, suggesting enhanced RIPK3/ZBP1-mediated necroptosis (78). Yet another cell type associated with psoriasis pathogenesis are T cells (**Figure 3**). T cell activation, cytokine production, and inflammation can contribute to abnormal immune responses in psoriasis – all of which can be regulated by RIPK1 scaffolding function and kinase activity (**Figure 3**). Indeed, inhibition of RIPK1 kinase activity significantly reduced necroptosis and inflammatory responses, as evidenced by decreased levels of IL-1β, IL-6, IL-17A, IL-23a, CXCL1, and CCL2, suggesting that RIPK1 can impact T cell-dependent immune cell activation and monocyte and neutrophil chemotaxis (79) (**Figure 3**). Although direct evidence of RIPK1’s role in stromal cells within psoriatic tissue is limited, its known involvement in regulating cell death in epithelial cells suggests that RIPK1 may contribute to tissue homeostasis beyond T cell activation (79). This broader influence likely plays a role in shaping psoriatic disease pathology. These data support an association between low RIPK1 expression and increased psoriatic disease severity. Clinical studies have evaluated a RIPK1 inhibitor (GSK2982772) in patients with mild-to-moderate plaque psoriasis (80, 81) (**Table 1**). Despite the authors stating the achievement of near complete RIPK1 target engagement and reduction in inflammatory cytokines at 4 weeks, these changes did not translate into greater clinical benefit between the active treatment *vs* placebo groups (82). Notably, this clinical study had a small sample size of 52 patients, thus limiting statistical power when comparing treatment groups (82). While the current findings support minimal diminished cytokine expression, the changes in disease activity were not statistically analyzed; therefore, the findings in this study may be insufficient to accurately assess the therapeutic potential of targeting RIPK1 in psoriasis.

### 4.4 Inflammatory bowel disease

**Figure 4:**
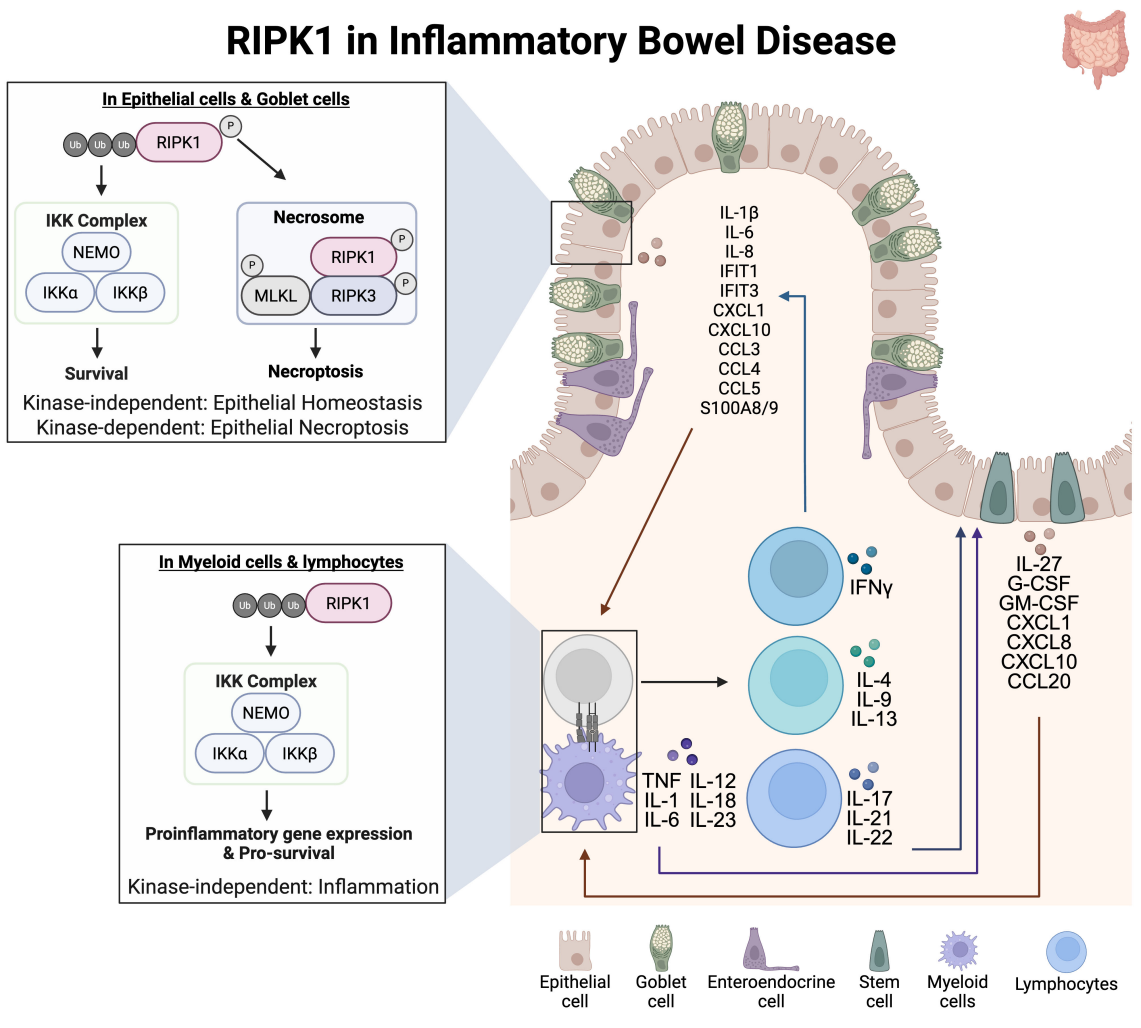
RIPK1 in inflammatory bowel disease. The complex IIb spontaneously forms in epithelial cells overexpressing cFLIP. As cFLIP blocks activation of caspase 8, the complex IIc evolves into the necrosome, resulting in oligomerization of MLKL and subsequent membrane permeabilization. Necroptotic cells of intestinal epithelial cells (IEC) release cytokine and chemokines such as IL-27, G-CSF, GM-CSF, CXCL1, CXCL8, CXCL10, and CCL20, that subsequently activate nearby myeloid cells: macrophages or dendritic cells (antigen presenting cells, APC), resulting in pro-inflammatory cytokine release. IL-1β damages the intestinal epithelial cells and goblet cells thereby disrupting barrier function. TNF signaling on damaged IELs can induce necroptosis and lead to secretion of necroptosis-associated factors. In addition to the release of necroptotic markers, IECs in response to IL-1β produce chemokines that recruit lymphocytes and myeloid cells to the site of inflammation. Recruited lymphocytes are activated by IL-1β and release IL-22 that acts on IECs, resulting in cell necroptosis and mucosal damage. Ultimately, necroptotic IECs can further potentiate inflammation.

There is growing evidence supporting the association between dysregulated RIPK1 and IBD. In human patients, missense mutations within the death domain of RIPK1 can result in IBD-like symptoms, such as diarrhea, abdominal pain, and weight loss, suggesting an association between RIPK1 and symptoms associated with IBD (55, 56, 73). It is possible that the loss of function of the death domain of RIPK1 results in a combined immunodeficiency attributed to altered cytokine levels and necroptotic markers such as, IL-1β, IFIT1/3, CXCL1. CXCL10, and S100A8/9 (**Figure 4**). Despite the growing association of RIPK1 in the pathogenesis of IBD, the role of RIPK1 remains controversial. For instance, mice with intestinal epithelial cell-specific RIPK1 deficiency develop colitis and show disrupted tissue architecture due to increased epithelial cell death (**Figure 4**), suggesting that RIPK1 has a protective role in regulating gut epithelial homeostasis (3, 65). However, RIPK1 also exhibits a dual function in intestinal epithelial cells, where its scaffolding function supports barrier integrity, while its kinase activity can promote inflammation and cell death (65). RIPK1 deficiency was introduced through transgenic manipulation, affecting both its scaffolding function and kinase activity (65). These findings suggest that inhibiting RIPK1 kinase activity with a RIPK1 inhibitor could preserve epithelial function while retaining homeostatic signaling mediated by scaffolding activity. Supporting this, administration of a RIPK1 inhibitor, a kinase-specific inhibition of RIPK1, in a DSS-colitis murine model resulted in improved intestinal barrier homeostasis by reducing epithelial monolayer disruption and maintaining epithelial permeability (**Figure 4**), suggesting that RIPK1 can contribute to epithelial cell death (56, 83). These findings support inhibiting RIPK1 kinase activity to regulate intestinal homeostasis. Research in murine models of IBD and phase I clinical trials has shown that RIPK1 inhibitors did not result in severe adverse drug reactions (56, 83, 84). These seemingly opposing roles of RIPK1 are likely attributed to cell type-specific effects.

### 4.5 Neuroinflammation

**Figure 5:**
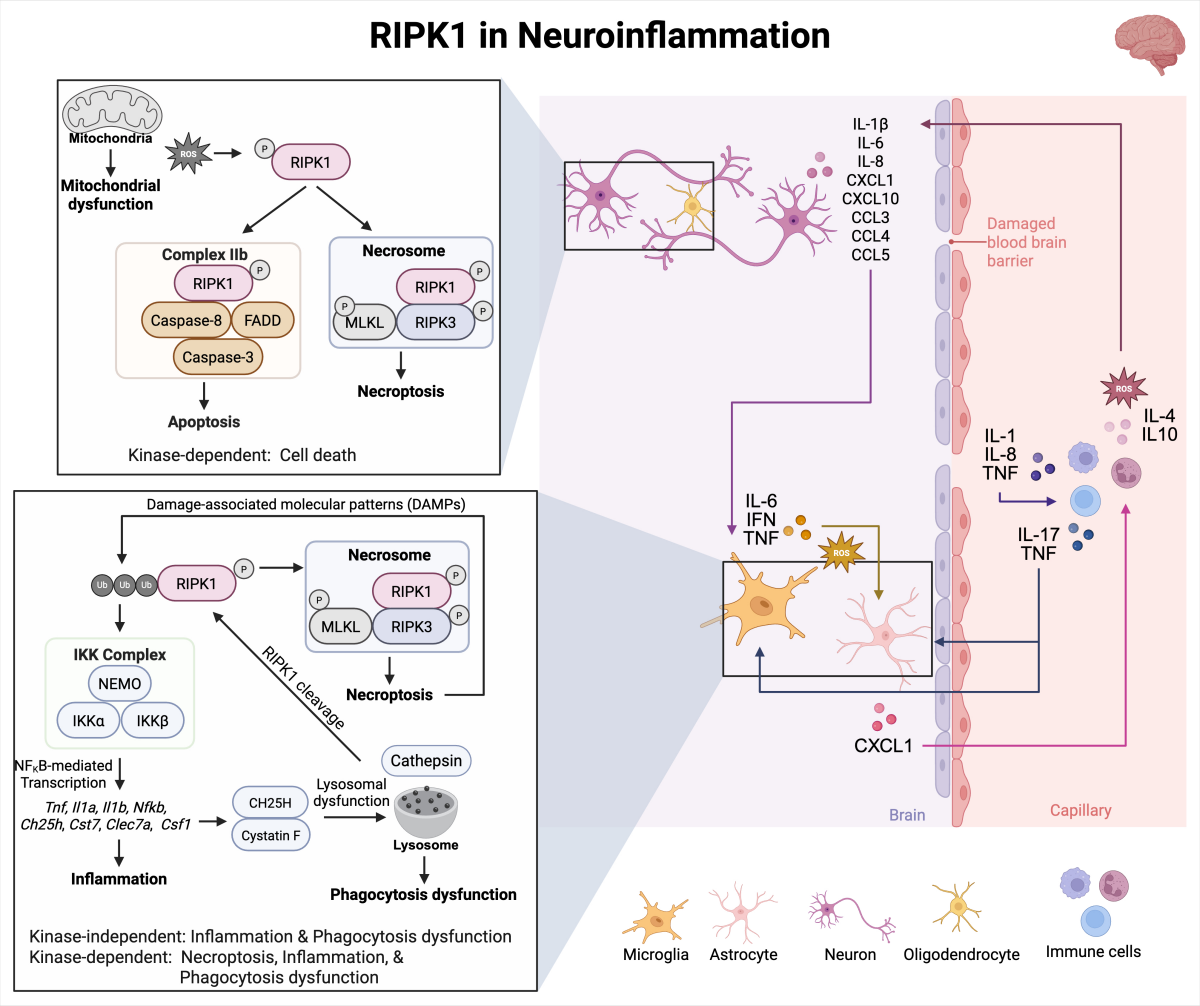
RIPK1 in neuroinflammation. In neuroinflammatory diseases, RIPK1-dependent apoptosis and necroptosis cause the release of DAMPs that can activate the NF-κB pathway to promote secretion of necroptotic markers. These markers can activate several pathways involving microglia, astrocytes, and immune cells. RIPK1 kinase activity and scaffolding function contribute to microglial and astrocyte cell death. Activated RIPK1 and ROS generation can enhance each other in a positive feedback loop. Excessive production of ROS can activate RIPK1 downstream signaling pathways and increase RIPK1 kinase activity. Furthermore, RIPK1 activation promotes ROS production by enhancing phosphorylation of the pyruvate dehydrogenase complex.

RIPK1 is broadly expressed in all major cell types in the CNS, including neurons, microglia, astrocytes, and oligodendrocytes (85). Its kinase activity has been implicated in the pathogenesis of several neuroinflammatory and neurodegenerative diseases, such as amyotrophic lateral sclerosis (ALS), acute ischemic brain injury (86), MS (64, 87), and Alzheimer’s disease (70). RIPK1 can regulate cell fate decisions, such as apoptosis, necrosis, and inflammation (68). In ALS, elevated RIPK1 expression and kinase activity in spinal cords drive inflammatory astrocyte and microglia activation (85). Inhibition of RIPK1 in murine models of ALS delays disease progression and reduces neuroinflammation (85). Similarly, in murine models of cerebral ischemia, RIPK1 activation in microglia and astrocytes leads to the release of proinflammatory cytokines and lysosomal dysfunction, respectively, while in neurons and oligodendrocytes, RIPK1 activation contributes to cell death (88, 89) (**Figure 5**). The inhibition of RIPK1 has been shown to protect against oligodendrocyte degeneration in a murine model of motor dysfunction (69), underscoring its role in axonal degeneration and neuroinflammation (**Figure 5**). Moreover, both *in vitro* models of oxidative stress and *in vivo* models of cerebral ischemia have shown pharmacological inhibition of RIPK1 with Necrostatin-1 (Nec-1) significantly attenuates neuronal necroptosis, supporting its therapeutic potential in reducing neuroinflammatory damage (88, 89) (**Figure 5**).

Importantly, it remains uncertain whether differential signaling occurs downstream of RIPK1-activating stimuli. However, it is possible that this harmful environment is perpetuated by activated microglia, astrocytes, and infiltrating immune cells like T cells and macrophages, through the release of disease-associated inflammatory mediators, such as IL-1β, IL6, IL8, CXCL1, CXCL10, CCL3, CCL4, and CCL5 (**Figure 5**). Thus, RIPK1 not only triggers inflammatory cell death but also enhances inflammatory signaling, creating a feedback loop that accelerates neurodegeneration (70). Therefore, targeting the kinase activity of RIPK1 may serve as a noteworthy therapeutic approach for treating chronic inflammatory diseases of the CNS. This can be achieved by safely modulating RIPK1 to simultaneously inhibit various harmful mechanisms that contribute to neurodegeneration.

## 5 Clinical assets

Despite RIPK1’s potential as a therapeutic target for a wide range of diseases, there are no approved RIPK1 inhibitors currently on the market. Nec-1, the first small-molecule RIPK1 inhibitor described in the literature, was discovered by screening a compound library for inhibitors of TNF-induced necrosis in human monocytic U937 cells (86). Nec-1 and a more stable analog Nec-1s have proven to be valuable tools for studying the role of RIPK1 in disease models (86). However, poor pharmacokinetic properties, including half-lives of approximately 1 to 2 hours (90, 91), have limited their development as drugs. Within the past decade, several more promising RIPK1 inhibitors have been evaluated in clinical trials for various diseases (see **Table 1**). These agents are designed to selectively inhibit RIPK1’s kinase activity while preserving its scaffolding function, thereby minimizing disruption of essential survival and inflammatory signaling pathways.

### 5.1 GlaxoSmithKline – GSK2982772

GSK2982772 is a RIPK1 inhibitor that was being developed by GlaxoSmithKline (GSK) for treatment of chronic inflammatory diseases. Three phase 2 clinical trials were conducted: one in patients with mild-to-moderate psoriasis (81), one in patients with moderate-to-severe RA (92), and one in patients with active ulcerative colitis (82). GSK2982772 has a short half-life (2 to 3 hours), which requires 60-mg dosing orally 2 or 3 times daily. Although it was well tolerated, GSK2982772 did not demonstrate meaningful clinical improvements compared to placebo in patients with RA or active ulcerative colitis treated for 12 weeks. In patients with plaque psoriasis, treatment with GSK2982772 led to reductions in epidermal thickness and T-cell infiltration of psoriatic skin compared with placebo. In addition, improvements in the plaque lesion severity score were observed with increasing tertiles of GSK2982772 trough concentrations (81). To examine whether efficacy could be improved with higher systemic exposures, a modified-release formulation of GSK2982772 was developed that allowed 960-mg dosing once daily. The new formulation was tested in a phase 1 trial of patients with moderate-to-severe plaque psoriasis (80) and increased the trough plasma concentrations by more than ten-fold. A modest reduction in circulating inflammatory cytokines was observed, but the proportion of patients who achieved the primary endpoint of 75% improvement from baseline in Psoriasis Area Severity Index (PASI75) score at Week 12 was similar between GSK2982772 and placebo. A clinical signal was observed at PASI50 (42% *vs* 20% placebo at Week 12) but was not enough to warrant continued clinical development of GSK2982772 (80).

### 5.2 Denali Therapeutics and Sanofi – DNL747/SAR443060

DNL747 (also known as SAR443060) is a brain-penetrant RIPK1 inhibitor that was being developed by Denali Therapeutics and Sanofi for treatment of neurodegenerative diseases. Two phase 1b clinical trials were conducted: one in patients with Alzheimer’s disease and one in patients with ALS (93). DNL747–200 mg twice daily was the initial dose selected for both studies but was lowered significantly after adverse events including thrombocytopenia, anemia, and bleeding were observed in a preclinical 3-month study in monkeys. In both the Alzheimer’s disease and ALS studies, dosing orally with DNL747–50 mg twice daily for up to 28 days was well tolerated and showed distribution of DNL747 to the cerebrospinal fluid. At steady-state trough concentrations, inhibition of RIPK1 in peripheral blood mononuclear cells was 81.83% in the Alzheimer’s disease study and 65.92% in the ALS study. The studies were not adequately powered to assess whether treatment with DNL747 led to meaningful clinical changes in the digital clock-drawing test for the Alzheimer’s disease study or in the Revised ALS Functional Rating Scale (ALSFRS-R) for the ALS study. Development of DNL747 was discontinued due to the potential risk of dose-limiting toxicities. Focus then shifted to DNL788 (also known as SAR443820), another brain-penetrant RIPK1 inhibitor that did not show similar dose-toxicity concerns. DNL788 was tested in a phase 2 clinical trial in patients with ALS but failed to meet the primary endpoint of meaningful improvement in ALSFRS-R. DNL788 was also tested in a phase 2 trial of patients with MS but failed to meet the primary endpoint of meaningful improvement in serum neurofilament light chain levels, a biomarker of neuronal degeneration.

### 5.3 Compounds currently in clinical trials

Current clinical trials are investigating several RIPK1 inhibitors, each at different stages of development, with a rationale rooted in RIPK1’s role in inflammation, immune response, and cell death. DNL758/SAR443122 (Denali/Sanofi) is being tested in phase 2 trials for moderate-to-severe ulcerative colitis. The rationale is based on RIPK1’s involvement in inflammatory signaling pathways, which are key drivers of ulcerative colitis. AC-003 (Accropeutics), in phase 1 trials, is under evaluation for treatment of acute graft-versus-host disease, where RIPK1 inhibition is hypothesized to reduce immune-mediated tissue damage. GDC-8264 (Genentech), also in phase 1 trials, is being assessed for its potential to treat patients at risk of cardiac surgery-associated acute kidney injury and major adverse kidney events. These conditions are associated with inflammation and tissue injury, processes driven by RIPK1-related pathways. Finally, LY3871801/R552 (Eli Lilly/Rigel), in phase 2 trials, is undergoing evaluation for treatment of active moderate-to-severe RA. In a first-in-human phase 1 study (EudraCT: 2019-002520-32), LY3871801/R552 exhibited a prolonged half-life and was well tolerated in healthy adult volunteers treated once daily for up to 14 days (84). RA is marked by chronic inflammation and immune activation, where targeting RIPK1 could suppress pathogenic inflammation and promote tissue homeostasis. These trials represent important steps in advancing promising RIPK1 inhibitors as therapeutic agents for inflammatory and degenerative diseases (84).

## 6 Discussion

Mutations in the RIPK1 gene have been linked to various diseases, including autoimmune disorders with established human causality. This underscores the potential for targeting RIPK1 as a promising therapeutic strategy in inflammatory diseases (56, 94). However, the diverse effects of RIPK1 across different tissues and diseases present challenges that must be addressed to fully leverage its therapeutic potential.

Despite the promising outlook, various drugs targeting RIPK1 have not been successful due to several factors. In the neuroinflammation space, the clinical dose of the RIPK1 inhibitor tested failed to achieve >90% target engagement (93). Target engagement may be essential for this target as serum exposure levels influence the achievement of full drug efficacy, minimize unintended interactions with other biological systems, and determine the optimal dosage for treatments. Limited sample sizes and high placebo response rates can also contribute to failures in clinical trials making it difficult to determine therapeutic efficacy and safety. Lastly, literature presented in this review supports a unique role for RIPK1 kinase activity to primarily regulate epithelial homeostasis, which is separate from its scaffolding functions associated with inflammation. Directly targeting the scaffolding function of RIPK1 is intentionally avoided, as such interventions could interfere with NF-κB signaling—a pathway whose disruption has been linked to safety concerns (95). Consequently, RIPK1 inhibitors may be more appropriately utilized within combination therapies alongside anti-inflammatory agents, thereby enabling a sequential approach: first attenuating the immune response and subsequently promoting tissue homeostasis. It’s likely that RIPK1 inhibitors might be better suited in combination therapies with anti-inflammatory targets to provide a sequential strategy for 1) dampening the immune response, and 2) restoring tissue homeostasis (96). Addressing these challenges through optimized clinical trial designs, robust combinational strategies, and better target engagement could enhance the clinical efficacy and safety profiles of RIPK1 inhibitors. A potential therapeutic strategy could involve biasing signaling towards complex IIa to promote apoptosis or blocking complex I while maintaining complex IIa function to prevent necroptosis.

Moving forward, the goal remains to develop effective and safe oral RIPK1 inhibitors. Gaining deeper insights into the interplay between RIPK1’s kinase-dependent and kinase-independent pathways will illuminate its role in disease mechanisms and aid in the discovery of predictive biomarkers for drug responses. These complexities necessitate further research to enhance our understanding of RIPK1’s functions and refine therapeutic approaches. Combining RIPK1 inhibitors with other therapies holds promise for treating a range of conditions by providing a more comprehensive approach to treating inflammation by harnessing the strengths of multiple drugs to address both inflammation and tissue health.
